# Update: Vitamin B12 Deficiency Among Bhutanese Refugees Resettling in the United States, 2012

**Published:** 2014-07-18

**Authors:** Kendra Cuffe, William Stauffer, John Painter, Sharmila Shetty, Jessica Montour, Weigong Zhou

**Affiliations:** 1 Division of Global Migration and Quarantine, National Center for Emerging and Zoonotic Infectious Diseases, CDC; 2 Division of Infectious Diseases and International Medicine, University of Minnesota Medical School; 3 Refugee Health Program, Texas Department of Health Services

In 2008, clinicians performing routine medical examinations in the United States reported high rates of hematologic and neurologic disorders caused by vitamin B12 deficiency in resettled Bhutanese refugees (1). To confirm this finding, CDC screened Bhutanese refugees’ serum samples for vitamin B12 levels and found vitamin B12 deficiency in 64% (n = 99) of samples obtained before departure and 27% (n = 64) of samples obtained after arrival in the United States (1). In response, CDC recommended that arriving Bhutanese refugees receive oral vitamin B12 supplements and nutrition advice (1). In 2012, based on anecdotal reports of decreasing rates of vitamin B12 deficiency in this population, CDC worked with select domestic refugee health programs to determine if the recommendations had reduced the vitamin B12 deficiency rate among Bhutanese refugees.

All refugees who underwent medical screening at the Austin-Travis County clinic in Texas received the previously recommended interventions. Nutrition surveys were administered to measure dietary practices and consumption, overseas and post-arrival, of foods rich in vitamin B12 (e.g., red meat and eggs). The surveys were administered, nutritional counseling was provided, and vitamin B12 serum concentrations were measured pre-intervention and 3 months post-intervention.

A total of 49 Bhutanese refugees aged ≥18 years were included in the assessment. The median age of enrollees was 29 years (range = 17–65 years). Two (4%) were deficient at baseline. The median serum concentration pre-intervention was 344 pg/mL (range = 138–718 pg/mL). Only four (8%) of those screened had any knowledge of vitamin B12.

After intervention, vitamin B12 serum concentrations improved in 47 (58%) of enrollees, all of whom had normal serum concentration levels (normal range = 203–900 pg/mL), and the median serum concentration increased from 344 pg/mL to 402 pg/mL (range = 129–1,746 pg/mL). The two refugees found to be deficient pre-intervention were not deficient post-intervention and had serum concentrations of 276 pg/mL and 260 pg/mL. Two (4%) new cases were detected at follow-up. Improved knowledge of vitamin B12 was demonstrated by 28% of enrollees, and 85% reported consuming more foods rich in vitamin B12. Of the refugees reporting a change in dietary habits post-arrival, 40 (84%) cited improved availability of vitamin B12 rich foods.

Ongoing screening of newly arrived refugees in Minnesota also demonstrated an overall reduction in B12 deficiency post-implementation of the 2011 recommendations. Of 326 Bhutanese refugees screened in Minnesota on arrival, 38% (n = 84) were deficient in 2010, compared with 28% (n = 122) in 2011 and 17% (n=143) in 2012.

Although findings are preliminary, providing nutrition advice and vitamin B12 supplementation to resettled Bhutanese refugees, coupled with improved access to foods rich in vitamin B12, likely resulted in increased vitamin B12 serum concentrations. In addition, the rate of vitamin B12 deficiency might be decreasing among Bhutanese refugees in the refugee camps, possibly because of improved food supply in the camps and increased financial assistance from resettled refugees to those awaiting resettlement. Nonetheless, vitamin B12 deficiency remains high in Bhutanese refugees. CDC recommends that domestic refugee health programs provide Bhutanese refugees with oral vitamin B12 supplements and nutritional advice on arrival (1).
